# Mr. Stevenson, on Poisonous Plants

**Published:** 1805-11-01

**Authors:** John Stevenson

**Affiliations:** Surgeon. Kegworth


### *To the Editors of the Medical and Physical Journal.*

"*Cicuta Homini venenum est.*" PLINY.

GENTLEMEN,

NOTWITHSTANDING a *concise* statement of the following interesting particulars has recently appeared in the Nottingham Journal, I presume to think with many of my judicious friends, that the cause of philanthropy will be essentially promoted, by giving it, in an enlarged and more correct form, that degree of publicity, which your widely circulating Miscellany is calculated to afford.

Poisonous ingredients are sometimes incautiously conveyed into the constitution, through the medium of substances otherwise the most innocent and salubrious. That grateful beverage cider becomes highly pernicious by being impregnated with a preparation of lead. Even common water, according to some late and very ingenious experiments, is found to contract a similar contamination from passing through a *leaden* pump, and becomes consequently more or less detrimental to the health. But do many imagine that *common parsley* can be the vehicle of that dangerous narcotic herb, lesser hemlock? Yet, of the possibility of this, two ladies of Castle-Donnington, Leicestershire, were lately unfortunate examples. The hemlock was eaten with some sallad, wherein it had been put by mistake with common parsley, for which it had grown and been gathered. Symptoms of an alarming kind soon followed, indicative of the full operation of that pernicious vegetable. Had the mistake remained undiscovered, there is a just ground for a strong presumption, that the errror would have produced the most tragical event. I will here briefly enumerate the symptoms which supervened, in order that any person casually labouring under them, may be enabled; by comparison, to ascertain their real origin.

A troublesome nausea with occasional vomiting occurred, accompanied with oppressive head-ach and giddiness; also a strong propensity to slumber, at the same time that *calm* repose was wholly prevented by frequent startings, and excessive agitations. The mouth, throat, and stomach, were impressed with the sensation of a pungent heat, attended with great difficulty in swallowing. Increased thirst prevailed, with total loss of appetite of every kind of solid aliment The extremities felt benumbed, and and were affected with tremors; and all the vital and animal functions were performed with unusual inactivity.

It may not be deemed uninteresting, by way of illustration, to state in this place, the different and opposite sentiments entertained by several eminent botanical writers, relative to the effects resulting from the incautious use of this plant. It is curious enough, that some authors altogether overlook it, as Turner, and the Editors of the Encyclopædia Britannica, &c. Gerard, Ray, Parkinson, and Hill, after describing the cicuta minor, take no notice of its poisonous quality, although they all speak of it as being *liable* to be mistaken for parsley, yet without adducing any instances of the kind. Others again speak of it *cursorily*, as an herb capable of exciting slight and transient inconveniences; and there are not wanting characters of the first respectability, who allege that it is a *violent narcotic poison*. Thus, e. g. "We are not sure," says Sowerby, "of the poisonous qualities attributed by some to this plant, but it is at *least unwholesome* as well as unpleasant, and by no means eligible for food." Withering observes, "that this plant, from its resemblance to parsley, has sometimes been mistaken for it; and when eaten, it occasions *sickness*."

To conclude with authorities of its unquestionably deleterious properties, I select the subsequent extract upon this plant from 2 tom. p. 256, of Elementaires de Botanique. "Toute la plante de la *petite cigue* a une saveur *d'ail*; elle est nausieuse, resolutive, calmante interieurement; c'est une caustique tres dangereux à l'exterieur. Elle ne se mele que trop souvent avec *l'herbage*. On n'emploiè que *l'herbe*. On pourroit dans *le besoin la substituer* a la *précédente*: i. e. The conium maculatum, the effects of which are well known to be dangerously narcotic. Dr. Willich has the following passage in his Domestic Encyclopædia, under the article fool's-parsley. This noxious weed greatly resembles common parsley, for which it is sometimes mistaken, and when eaten with other plants, it occasions *vomiting, violent cholic*, and other *painful symptoms*."

Lightfoot, in his Flora Scotica, vol. 1. p. 165, furnishes the following very apposite quotation. "This plant (the lesser hemlock) is of a poisonous nature, producing *stupors, vomitings*, and *convulsions*. Cooks therefore cannot be too careful that they mistake it not for *parsley*, which it a good deal resembles." Chambers, in fifth Edition Cyclopædia, declares, "that the *lesser* hemlock is not less *dangerous*

*gerous* than the *greater*; it is even supposed *more violent* as well as more *hasty* in its *operation.* Some persons have been rendered *delirious* by eating porridge, wherein it had been used instead of parsley." Again, Bauhin, in 3 tom. p. 180, Historia Plantarum, Venenosam et perniciosam esse plantam cicutariam (cicutam minorem) testatur Dalechampius dicens; Esû cicutæ quæ apii hortensis specie incautum fefellerat, ego quendam novi ad *extremum usque vitæ dementem factum.*" Lastly, Mr. P. Miller, in his Dictionary, expressly remarks, "that it is so like *parsley*, that some unskilful persons have gathered and used it as such, by which *several* persons have suffered in their health, and *some* have been *destroyed* thereby."

To what cause must we attribute, and how reconcile these discordant opinions? I think there is good ground for believing, that the lesser hemlock is a plant with which every writer is not acquainted, owing perhaps to the insignificant rank it has held hitherto in the catalogue of poisons. And of the number of those who have given its character from the different effects ascribed to it, we may fairly doubt, whether many of those have actually had an opportunity of observing its genuine operation upon the human system. These, of course, would transcribe only the sentiments of other anthors upon this subject, with which they might casually meet. I must add too, that the reports of those who might witness its deleterious power would necessary vary according to the phenomena; and it is well known, that poisons, taken in the same proportions, exert (in consequence of peculiar idyosyncracies) very various degrees of violence upon different individuals, comprehending a gradation from the slightest symptoms that would scarcely arrest attention to those which are most alarmingly dangerous and even fatal; and it may be further observed, that the poisonous qualities of a plant are materially modified by the *season*, and especially by the peculiar *soil* in which it grows; for a moist shady situation probably will render the *lesser hemlock* both more luxuriant as well as more virulent. Thus we find the cicuta aquatica (another species of this plant) grows *only* in *wet* places, which is amongst the most *poisonous* plants that this country affords; the fatal juice of which, indeed, is supposed by some to have been exhibited to the great Athenian philosopher. Nor is it irrelevant to add, that the lesser hemlock with which the two ladies above stated were so seriously affected, grew in a garden almost secluded ed from the penetrating solar rays, by spreading trees, and surrounding buildings.

Another source also of this diversity of sentiment may partly have arisen from the *variety* of appellations which have been affixed to the subject of these remarks. Linnæus, Withering, Sowerby, &c. denominate it æthusa, aquapium; Parkinson, cicuta minor seu fatua; Miller, cicuta minor, &c. Ray and Gerard, cicuta tenuifolia; Lobel, cicutaria fatua; Thalius, apium cicutarium, tabermontanus petroselinum caninum; Gesner, apii comes vitium, &c. Hence, perhaps, some, not intimately acquainted with the distinctive characteristics of the *lesser hemlock*, have confounded it with the cicutaria vulgaris, *bastard hemlock*, which Mr. Parkinson says, grows only in gardens in these parts, and is indeed not unlike it in appearance, though not so poisonous. It is not impossible also, that the charophylum sylvestre, named *small hemlock* by Ray, may, by those who are not conversant in botanical criteria, have been mistaken for the plant in question. In this case, as the leaves, in times of scarcity, are sometimes made use of with impunity as pot herbs in some parts of this kingdom, the effects produced, would be described as the most remote from those of a narcotic origin.

It is truly singular, that though all writers upon this weed mention its very close analogy to common parsley, yet no description, sufficiently characteristic of their *respective particularities*, is annexed, to enable a person not a professed botanist, to recognize each individually; e. g. Sowerby says, it may be distinguished by its dark dull green leaves, and *garlic* smell. Withering, on the contrary, is *silent* respecting the *colour* of the leaves, but intimates that they are glossy; and though several authors speak of the *garlic* odour of this plant, yet I agree with Ray and Bauhin on this point, who decidedly deny its possessing *any such smell*, by the words *"odore nullo."* In short, without enumerating the contradictory descriptions to be found in different writers upon this plant, I shall only observe, that some glaring misrepresentation, or faulty omission, is visible in all the histories which I have had an opportunity of examining. As, however, it must be deemed a circumstance of the last importance to be furnished with infallible marks of distinction which exist at all times in these respective vegetables, I will endeavour, by contrasting the two, to point out such as will enable any one invariably to detect the *lesser hemlock* when found mixed with common parsley. I must here observe, that it is during their *early* growth, that the striking resemblance obtains. Then indeed the similarity is so close as easily to deceive any one not intimately acquainted with their peculiar differences. Hence it is, that the *lesser hemlock* has acquired the significant epithet of fool's parsley. Even at this period they may be distinguished with a degree of certainty, by the *round, branched,* and *hollow* stem of the *lesser hemlock,* rising *singly* from its root, having a violet tinge on the side exposed to the sun, (but without *maculæ,* or *spots,* as in the common hemlock, conium maculatum Linnæi); whereas *parsley* grows from the bottom of the plant, the first year in *several* long *leaved-stalks.* The leaves also of this hemlock are finer, more acute, decurrent, and of a darker hue. But the difference of their *smell,* and especially of their *taste,* at once establish their characters. It is an *annual* plant, the parsley on the contrary flowers only the *second* year. Should this hemlock have grown into flower *unobserved* with the parsley, still invariable characteristics would present themselves, which should studiously be regarded, in order that the *lesser hemlock* may be eradicated, to prevent its propagating itself by means of its seeds. I allude to three *long* fencelets, consisting of pendant leaves going *half* round *each rundle,* which in parsley are *wanting,* and in lieu thereof, there is a fence of short rising leaflets *surrounding* the umbels. The *flowers* too of the *lesser hemlock* are *white,* those of parsley *yellow.* Each flower of the former, terminates in roundish scored seeds, of the latter in two semi-convex ones, adhering by a *flat* surface to each other. If to these criteria be added the *smell* and *taste,* it is scarcely possible to commit a mistake.

From what has been already advanced, we may, I apprehend, without fear of contradiction, infer, that the *lesser hemlock,* though hitherto very confusedly treated of, is *really* an *actively poisonous herb*; and as it is by *no means* an unfrequent intruder amongst parsley, for which it is unquestionably liable to be mistaken, it doubtless deserves to have its character, and its noxious qualities, universally known and exposed. The slight effects consequent upon a small quantity *only* being consumed, have perhaps occasioned it to be overlooked so very commonly. Hence a dangerous poison is unconsciously used, which insiduously undermines the health. For it is not at all improbable, that indigestion, or other latent causes of indisposition, derive their origin from this source much more frequently than is supposed. And as it is in common with parsley, not not seldom applied to culinary purposes, its pernicious influence may probably be much diminished, though not wholly destroyed, by this means. It is, I presume, in its *crude native* state, (as in the examples already detailed) that it exerts its full and destructive energies. I will conclude these remarks by strenuously recommending the *curled* parsley, apium crispum, to be cultivated instead of the *common,* as it is not only much more *elegant,* but also possesses the same virtues, and is so widely different in appearance from the lesser hemlock, as almost to preclude the possibility of mistake. I am, &c.

JOHN STEVENSON, Surgeon.

*Kegworth, Sept.* 7, 1805.

